# Association between phosphorus intake and bone health in the NHANES population

**DOI:** 10.1186/s12937-015-0017-0

**Published:** 2015-03-21

**Authors:** Albert W Lee, Susan S Cho

**Affiliations:** 1 NutraSource, Royal Oak, MI USA; 2 NutraSource, 6309 Morning Dew Ct., Clarksville, MD 21029 USA

**Keywords:** Phosphorus intake, Bone mineral content, Bone mineral density, Osteoporosis

## Abstract

The objective of this study was to estimate the independent associations between intake of phosphorus (P) and bone health parameters such as bone mineral content (BMC) and bone mineral density (BMD). It provides odds ratio (OR) of osteoporosis with quartiles of P intake adjusted for covariates (i.e., age, gender, BMI, and consumption of calcium (Ca), protein, total dairy foods, and vitamin D as well as intakes of supplemental Ca, vitamin D, and multivitamins/minerals). Data came from males and females aged 13–99 years who participated in the 2005–2010 National Health and Nutrition Examination Survey (NHANES). Analyses showed that higher P intake was associated with higher Ca intake, and that dietary Ca:P ratios (0.51-0.62, with a mean of 0.60 for adults) were adequate in all age/gender groups. High intake of P was positively associated with BMC in female teenagers (Q4 vs. Q1: BMC, 30.9 ± 1.1 vs. 29.0 ± 0.5 g, P = 0.001). It was also positively associated with BMC and BMD as well as reduced risk of osteoporosis in adults >20 years of age (Q4 vs. Q1: OR of osteoporosis, 0.55; 95% confidence interval [CI], 0.39- 0.79; P = 0.001; BMC, 37.5 ± 0.4 vs. 36.70 ± 0.3 g, P < 0.01; BMD, 0.986 ± 0.004 vs. 0.966 ± 0.005 g/cm^2^, P < 0.05). The data suggest that high intake of P has no adverse effect on bone metabolism in populations with adequate Ca intake, and that it is also associated with positive bone parameters in some age/gender groups.

## Introduction

Maintenance of healthy bones throughout the lifespan is physiologically beneficial. Greater bone formation and/or less bone resorption lead to an increase in BMC and/or BMD. Assessment of BMD is used to define osteoporosis, a systemic skeletal disease characterized by low BMD and abnormal bone micro-architecture. These deficits lead to fragility and increased susceptibility to fractures [1]. BMC and BMD are considered good biomarkers for bone health [2]. The main factors that affect maximum bone mass and strength are genetic predisposition and dietary habits [3]. Dietary interventions (e.g., Ca supplementation) increase BMD or limit loss of it in older adults and postmenopausal females, reducing the risk of osteoporotic fractures.

The effects of high P intake on bone parameters have been controversial. Many studies report that high P intake is harmful to bone health in subjects whose dietary Ca:P ratio is extremely low [4-10]. However, there is strong evidence that high P intake has no negative impact on Ca balance in normal subjects with appropriate dietary Ca and P intakes [11-15].

We hypothesized that in those with adequate Ca intake, the adverse effects of higher P intake on bone metabolism would be negligible. Therefore, we examined the association between P intake and bone health parameters in a cross-sectional cohort of the 2005–2010 NHANES.

## Methods

All analyses were completed using Statistical Analysis Software (SAS) 9.2 and SUDAAN version 11 and used sampling weights, primary sampling units and strata information provided by NHANES. Day 1 dietary, exam, lab and questionnaire data were used for NHANES 2005–2010 with exclusions for age < 13, incomplete or unreliable data and pregnant or lactating females. All analyses were done for males, females and genders combined. The age groups for the analyses were 13–19, 20–49, 50–99 and 20–99. The Osteoporosis questionnaire (OSQ) files were used to indicate presence of osteoporosis. The Prescription Medication (RXQ) files were used to identify subjects taking osteoporosis or estrogen medications. The Dual Energy X-ray Absorptiometry-Femur files were used for BMD (g/cm^2^) and BMC (g) measurements.

Phosphorus quartiles were calculated by age group and gender using SUDAAN proc descript. Means by phosphorus intake quartile for nutrient intakes, questionnaire responses and lab results were calculated using SUDAAN proc regress with separate regressions within age groups and gender using indicator variables for the P intake quartiles.

Least Square Means (LSM) and standard errors (SE) were calculated for total femur BMC and BMD across the quartiles of phosphorus intake using SUDAAN proc regress by age groups and gender. P-value statistics were calculated for quartile trend on quartile numbers on phosphorus intake in grams. The LSM analysis was done after adjustments for age, gender, energy, body mass index (BMI), protein intake, diary intake, calcium intake, vitamin D intake and flags for supplemental intake of calcium, vitamin D, and multi-vitamins. Flags were also included for taking osteoporosis medication and, for females, taking estrogen medication.

Odds ratios were calculated for the indication of osteoporosis across the quartiles of phosphorus intake using SUDAAN proc rlogist by age groups and genders. The same trend statistics were calculated as in the LSM analysis. The covariate sets used were as used in the LSM analysis except the flag for osteoporosis was not included. The 13–19 age group was not used in this logistic analysis since the NHANES questionnaire for osteoporosis does not include this age group. Two flags were used as indicators of osteoporosis. These were based on the prescription medication rx_drug files and the osteoporosis questionnaire osq files. Osteoporosis was determined as indicated if one or more of 2 conditions were satisfied: (1) Answer yes to question “Has a doctor ever told you that you had osteoporosis, sometimes called thin or brittle bones?” and (2) Answer yes to question “Has a doctor ever told you that you had broken or fractured your hip?”.

## Results and discussion

The importance of Ca and P in maintaining bone health is well established [3]. Based on Ca retention from balance studies, factorial estimates of requirements, and data on changes in BMD and BMC, the Institute of Medicine (IOM) [3] set an adequate intake (AI) of calcium at 1,200 mg/day for adults >50 years of age and a Tolerable Upper Intake Level (UL) at 2,500 mg/day. The Recommended Dietary Allowances (RDA) and UL for P are 700 and 4,000 mg/day for adults, respectively, and 1,250 and 4,000 mg/day in teenagers [3].

### Characteristics of the NHANES population and intakes of P and Ca

Table 1 shows characteristics of the NHANES population. Compared with teenagers and adults aged 20–49 years, those aged ≥50 years took in less dietary Ca and P, soft drinks, and dairy foods. They also used more supplemental Ca and multivitamins. The LSM of various ethnic groups were compared for intakes of Ca and P. Whites had the highest intakes of both Ca and P; Blacks had the lowest (Whites vs. Blacks: Ca, 1,013 vs. 799 mg/day, P < 0.01; P, 1,409 vs. 1,188 mg/day, P < 0.01; data not shown in tables).

**Table 1:** **Demographics of NHANES subjects**

Age group	13-19 years	20-49 years	50-99 years	20-99 years
Phosphorus (mg)	1382±192^a^	1443±14^b^	1259±20^c^	1368±11
Calcium (mg)	1070±19^a^	1020±13^b^	888±17^c^	966±10
Energy (kcal)	2255±27^a^	2309±21^b^	1896±28^c^	2147±16
Total Dairy (oz equivalent)	2.1±0.05^a^	1.8±0.04^b^	1.4±0.04^c^	1.6±0.0
Soft Drinks (oz)	14.9±0.7^a^	17.2±0.6^b^	9.6±0.43^c^	14.0±0.4
Supplemental Calcium (%)	20.8±1.2^a^	40.2±0.8^b^	56.6±1.6^c^	46.9±0.7
Multivitamin use (%)	18.8±1.3^a^	32.0±0.9^b^	51.1±1.7^c^	38.8±0.8
Osteoporosis medication (%)	NA	0.2±0.06^a^	5.3±0.58^b^	2.0±0.15

NA=not applicable. ^a,b,c^Different superscripts indicate statistically significantly different values from other age groups.

Table 2 shows that high P consumers (Q4) had P intake two to three times higher than the RDA. It was approximately 2.4 g/day, well below the UL of 4 g/day. None of the high P intake groups had P intake over the UL of 4.0 g/day. Data also showed that higher P intake was associated with higher Ca intake.

**Table 2:** **Phosphorus and Calcium intakes, mg/day**

Gender	LSM	Q1	Q2	Q3	Q4
Phosphorus intakes, mg/day					
13-19 yr, All	1382±14	597±7	1037±5	1471±8	2421±39
13-19 yr, Male	1624±23^a^	599±15	1055±8	1476±10	2484±49
13-19 yr, Female	1138±10^b^	596±8	1023±6	1465±10	2247±62
20-49 yr, All	1443±6	684±6	1121±3	1541±4	2426±21
20-49 yr, Male	1701±10^a^	718±9	1126±6	1548±5	2487±26
20-49 yr, Female	1179±4^b^	671±8	1118±5	1531±6	2231±32
50-99 yr, All	1377±4	701±6	1091±3	1451±4	2260±17
50-99 yr, Male	1592±8^a^	698±7	1087±5	1462±5	2315±23
50-99 yr, Female	1172±4^b^	703±7	1094±4	1438±5	2117±22
20-99 yr, All	1368±4	667±4	1071±2	1450±3	2283±14
20-99 yr, Male	1602±7^a^	693±6	1075±3	1457±4	2348±19
20-99 yr, Female	1145±4^b^	657±5	1068±2	1441±5	2110±21
Calcium intakes according to phosphorus intake quartiles, mg/day					
13-19 yr, All	1070±14	470±11	768±15	1144±20	1896±45
13-19 yr, Male	1243±23^a^	464±15	797±23	1114±28	1918±53
13-19 yr, Female	894.8±16^b^	472±14	747±17	1182±24	1833±83
20-49 yr, All	1020.5±7	496±9	791±12	1061±10	1732±25
20-49 yr, Male	1157±11^a^	477±14	746±19	1004±13	1742±30
20-49 yr, Female	880±8^b^	503±10	824±14	1137±16	1700±41
50-99 yr, All	895±6	462±9	710±8	946±13	1460±18
50-99 yr, Male	985±8^a^	437±11	678±11	888±11	1461±22
50-99 yr, Female	817±8^b^	472±10	730±11	1001±22	1460±29
20-99 yr, All	966±5	481±5	750±6	1010±8	1622±18
20-99 yr, Male	1086±8^a^	458±7	713±13	9529±9	1631±21
20-99 yr, Female	852±7^b^	490±6	775±8	1079±14	1597±27
Dietary Ca:P intake molar ratio					
13-19 yr, All	0.60	0.62	0.57	0.60	0.60
13-19 yr, Male	0.59	0.59	0.59	0.60	0.58
13-19 yr, Female	0.60	0.64	0.57	0.59	0.62
20-49 yr, All	0.55	0.57	0.54	0.53	0.55
20-49 yr, Male	0.52	0.51	0.52	0.50	0.54
20-49 yr, Female	0.58	0.61	0.56	0.56	0.59
50-99 yr, All	0.55	0.56	0.54	0.54	0.54
50-99 yr, Male	0.52	0.51	0.51	0.51	0.54
50-99 yr, Female	0.57	0.58	0.57	0.57	0.57
20-99 yr, All	0.55	0.57	0.54	0.54	0.54
20-99 yr, Male	0.52	0.51	0.51	0.51	0.54
20-99 yr, Female	0.58	0.60	0.57	0.57	0.58

^a,b^Different superscripts indicate statistically significantly different values between males and females.

Dietary intake of Ca was approximately 1,000 mg/day, with that among high P consumers 1.5-fold higher than the recommended RDA (1,620-1,890 mg/day). Ca intake was well below the UL of 2,500 mg/day [3]. None of the high P intake groups had Ca intake over the UL of 2.5 g/day. According to nationwide food consumption surveys, milk was the leading source of P as well as Ca in all age groups [16]. In all age/gender groups, NHANES data indicated that the dietary Ca:P molar ratio was adequate, with a range of 0.51-0.62. Compared with females, males had higher intakes of Ca and P (Table 2).

### Markers of bone metabolism and OR of osteoporosis

A multivariate model that included adjustments for intakes of calcium, protein, and vitamin D showed that high P intake combined with adequate Ca was associated with improved bone health. This was indicated by improved BMC, BMD, and/or reduced OR of osteoporosis in some age/gender groups. In all age/gender groups with higher Ca intake, increased P had no adverse effects on bone health parameters. The dietary Ca:P ratio was within the safe intake range. P intake was not associated with serum concentrations of vitamin D or P (data not shown). Compared with females, males had higher values for BMC and BMD (Tables 3 and 4).

**Table 3:** **Femur Bone mineral content (BMC) according to P intake quartiles**

Age, gender	LSM	Q1	Q2	Q3	Q4	P trend
13-19 yr, All	35.1±0.2	34.6±0.5	34.9±0.3	35.5±0.3	35.2±0.5	NS
13-19 yr, Male	40.1±0.3^a^	39.5±0.8	40.0±0.7	41.1±0.5	39.6±0.6	NS
13-19 yr, Female	29.6±0.2^b^	29.0±0.5	29.9±0.3	29.5±0.5	30.9±1.1	<0.01
20-49 yr, All	37.4±0.1	36.5±0.3	37.3±0.2	37.5±0.3	38.2±0.3	<0.01
20-49 yr, Male	43.8±0.2^a^	42.9±0.7	43.4±0.4	43.4±0.4	44.6±0.4	0.1010
20-49 yr, Female	30.6±0.1^b^	29.9±0.4	30.9±0.2	31.4±0.3	31.0±0.6	<0.05
50-99 yr, All	35.8±0.1	35.5±0.4	35.6±0.2	36.0±0.2	35.9±0.4	NS
50-99 yr, Male	43.6±0.3^a^	43.1±1.0	43.2±0.8	44.0±0.6	43.7±0.7	NS
50-99 yr, Female	28.6±0.1^b^	28.6±0.4	28.6±0.2	28.5±0.2	28.4±0.5	NS
20-99 yr, All	36.7±0.1	36.0±0.3	36.4±0.2	36.8±0.2	37.5±0.4	<0.01
20-99 yr, Male	43.7±0.1^a^	42.9±0.5	43.2±0.3	43.4±0.2	44.5±0.3	<0.05
20-99 yr, Female	29.7±0.1^b^	29.3±0.3	29.7±0.2	30.1±0.2	30.3±0.4	<0.05
Comparison of ethnicity; 20–99 yr, All (males and females combined)						
Hispanic	35.9±0.16^t^	35.7±0.32	36.1±0.31	35.8±0.20	36.0±0.32	NS
White	36.9±0.14^x^	36.0±0.28	36.5±0.23	37.0±0.21	37.5±0.25	<0.01
Black	38.2±0.21^y^	37.7±0.48	37.8±0.28	38.8±0.35	38.8±0.60	0.09
Other	33.5±0.33^t,z^	32.9±0.67	33.4±0.56	32.8±0.36	35.3±1.01	NS

Femur BMCs were calculated for each quartile after adjustments for age, gender, kcal, BMI, intakes of calcium, protein, total dairy foods, and vitamin D, and flags to denote supplemental calcium, supplemental vitamin D, supplemental multi vitamin/mineral and a flag to denote prescription medication for osteoporosis and estrogen medication (females only).

^a,b^Different superscripts indicate statistically significantly different LSM values between males and females.

^t, x, y, z^Different superscripts indicate statistically significantly different LSM values in 4 ethnic groups.

**Table 4:** **Femur Bone mineral density (BMD) according to P intake quartiles**

Gender	LSM	Q1	Q2	Q3	Q4	P trend
13-19 yr, All	1.000±0.003	0.990±0.01	0.999±0.006	1.003±0.005	1.009±0.008	NS
13-19 yr, Male	1.038±0.005^a^	1.027±0.02	1.039±0.013	1.048±0.008	1.035±0.010	NS
13-19 yr, Female	0.959±0.004^b^	0.945±0.011	0.963±0.006	0.956±0.011	0.993±0.021	NS
20-49 yr, All	1.014±0.002	0.999±0.006	1.016±0.004	1.017±0.005	1.023±0.006	<0.05
20-49 yr, Male	1.062±0.004^a^	1.058±0.012	1.058±0.006	1.056±0.006	1.069±0.008	NS
20-49 yr, Female	0.964±0.003 ^b^	0.948±0.007	0.970±0.005	0.977±0.007	0.971±0.010	<0.05
50-99 yr, All	0.924±0.002	0.919±0.006	0.923±0.005	0.927±0.004	0.926±0.007	NS
50-99 yr, Male	1.001±0.004^a^	0.996±0.009	1.005±0.009	1.004±0.006	0.998±0.008	NS
50-99 yr, Female	0.853±0.003^b^	0.858±0.008	0.849±0.006	0.852±0.006	0.852±0.011	NS
20-99 yr, All	0.976±0.002	0.966±0.005	0.973±0.004	0.977±0.004	0.986±0.004	<0.05
20-99 yr, Male	1.037±0.003^a^	1.034±0.008	1.036±0.005	1.029±0.004	1.044±0.005	NS
20-99 yr, Female	0.915±0.002^b^	0.909±0.005	0.912±0.004	0.922±0.005	0.924±0.008	NS
Comparison of ethnicity; 20–99 yr, All (males and females combined)						
Hispanic	0.997±0.003^t,z^	0.986±0.007	1.002±0.007	1.002±0.004	0.997±0.007	NS
White	0.964±0.002^t,x^	0.946±0.006	0.958±0.004	0.967±0.004	0.976±0.004	<0.01
Black	1.048±0.004^y^	1.041±0.008	1.043±0.005	1.054±0.007	0.968±0.010	NS
Other	0.944±0.005^z^	0.932±0.016	0.955±0.012	0.926±0.010	0.968±0.022	NS

Femur BMDs were calculated for each quartile after adjustments for age, gender, kcal, BMI, intakes of calcium, protein, total dairy foods, and vitamin D, and flags to denote supplemental calcium, supplemental vitamin D, supplemental multi vitamin/mineral and a flag to denote prescription medication for osteoporosis and estrogen medication (females only).

^a,b^Different superscripts indicate statistically significantly different LSM values between males and females or among different ethnic groups.

^t, x, y, z^Different superscripts indicate statistically significantly different LSM values in 4 ethnic groups.

Table 3 shows quartiles of the LSM of total femur BMC and a linear trend in P (mg). Data show a positive association between P intake and increasing BMC among those aged 20–99 years (3.4-4.2%; P < 0.05), in those aged 20–49 years (4.6%; P < 0.01), and females aged 13–19 years (6.6%; P < 0.01). Increases in P intake were associated with significant gains in total femur BMC. In males aged 20–49 years, there was an increasing trend of BMC in response to high P intake (4.0%; P = 0.10).

Of those aged 20–99 years, assessment by ethnic group attenuated statistical power for differentiation. Increased P intake was associated with higher BMC only in Whites (Q4 vs. Q1: 37.5 vs. 36.0; P < 0.01), with an increasing trend of BMC (Q4 vs. Q1: 38.8 vs. 37.7; P = 0.09) in Black participants. A comparison of LSMs showed that whites and Hispanics had significantly lower BMC than blacks, but it was higher than the level in other ethnic groups (including Asians).

Table 4 reports total femur BMD in each quartile. High P intake in those aged 20–99 years was associated with a 2.1% increase in total femur BMD. Similar increases were observed in all adults and females aged 20–49 years. In these groups, the highest quartile of P intake produced 2.0-2.4% greater BMD compared with the lowest quartile. In teenagers, P intake was not associated with BMD.

When those aged 20–99 years were compared by ethnicity, increased P intakes were associated with higher BMD only in Whites (Q4 vs. Q1: 0.976 vs. 0.946; P < 0.01). Whites and Hispanics had significantly lower BMD than blacks, but levels were higher than in other ethnic groups (including Asians). In adults aged ≥20 years, P intake was associated with improved bone health as indicated by increased BMD. In contrast, the Framingham Osteoporosis Study [17] found that total P intake was not related to BMD in 1,413 females and 1,125 males whose dietary Ca:P ratios ranged from 0.60 to 0.62.

Table 5 shows the OR of osteoporosis by quartile. Males and females aged 20–99 years with the highest P intake had a 45% lower risk of osteoporosis (OR = 0.55; 95% CI, 0.39-0.79; P < 0.01) compared with those who consumed the least P. When NHNANES subjects were divided into two age groups, 20–49 and 50–99, statistical power was attenuated. High P intake was associated with a decreasing trend for osteoporosis. Risk reduction was 54% (P = 0.10) in those aged 20–49 years. In those aged 50–99 years, it was 29% (P = 0.06).

**Table 5:** **Odd ratio of osteoporosis according to P intake quartiles**

	Odd ratio				
Gender	Q1	Q2	Q3	Q4	P trend ^ a ^
20-49 yr, All	1.00	0.39 (0.19, 0.78)	0.54 (0.26, 1.12)	0.46 (0.16,1.27)	0.10
20-49 yr, Male	1.00	0.36 (0.11, 1.20)	0.51 (0.16, 1.60)	0.60 (0.15, 2.38)	NS
20-49 yr, Female	1.00	0.37 (0.18, 1.00)	0.73 (0.29, 1.39)	0.27 (0.06, 1.33)	NS
50-99 yr, All	1.00	0.84 (0.63, 1.11)	0.77 (0.55, 1.08)	0.71(0.48, 1.04)	0.06
50-99 yr, Male	1.00	0.45 (0.23, 0.89)	0.55 (0.26, 1.15)	0.52 (0.17, 1.53)	NS
50-99 yr, Female	1.00	1.01 (0.71, 1.44)	0.98 (0.61, 1.59)	1.05 (0.52, 2.09)	NS
20-99 yr, All	1.00	0.76 (0.59, 0.97)	0.68 (0.51,0.91)	0.55 (0.39, 0.79)	<0.001
20-99 yr, Male	1.00	0.49 (0.30, 0.80)	0.56 (0.32, 0.97)	0.46 (0.22, 1.00)	0.08
20-99 yr, Female	1.00	0.85 (0.58, 1.24)	0.78 (0.44, 1.36)	0.65 (0.31, 1.38)	NS
Comparison of ethnicity; 20–99 yr, All (males and females combined)					
Hispanic	1.00	0.56 (0.61, 0.96)	0.61 (0.32, 1.14)	0.37 (0.15, 0.91)	0.07
White	1.00	0.82 (0.63, 1.07)	0.64 (0.39, 1.05)	0.67 (0.34, 1.32)	0.11
Black	1.00	0.69 (0.41, 1.18)	1.17 (0.63, 2.19)	1.31 (0.49, 3.54)	NS
Other	1.00	0.40 (0.11, 1.50)	0.27 (0.07, 0.97)	0.13 (0.01, 1.53)	0.05

Osteoporosis was determined as indicated if one or more of 2 conditions were satisfied: (1) Answer yes to question “Has a doctor ever told you that you had osteoporosis, sometimes called thin or brittle bones?” and (2) Answer yes to question “Has a doctor ever told you that you had broken or fractured your hip?”. ORs for osteoporosis were calculated for each quartile after adjustments for age, gender, kcal, BMI, intakes of calcium, protein, total dairy foods, and vitamin D, and flags to denote supplemental calcium, supplemental vitamin D, supplemental multi vitamin/mineral and prescription medication for osteoporosis and estrogen medication (females only).

^a^P trend values indicate statistically significantly different trends in 4 quartiles.

Increased P intake was associated with a lower OR of osteoporosis in various ethnic groups, with a decreasing trend in whites (OR=0.67; 95% CI, 0.34, 1.32; P = 0.11) and Hispanics (OR=0.37; 95% CI, 0.15, 0.91; P = 0.07). Other ethnic groups had a 87% lower risk of osteoporosis (P<0.05).

Several studies show that high P intake does not have a negative impact on Ca balance. Spencer et al. [11] found that an increase in P intake from 800 mg/day (the RDA) to 2,000 mg/day in adult males failed to affect Ca balance regardless of intake, which ranged from 200 to 2,000 mg/day. Similarly, Heany and colleagues [12-15] reported that intake of dietary P was inversely associated with both urinary Ca excretion and intestinal Ca absorption, implying a direct association with fecal Ca excretion. Changes in P intake by healthy adults affect Ca metabolism (i.e., decreased intestinal Ca absorption and decreased renal excretion of Ca). However, these outcomes most likely cancel each other out, leaving Ca balance unaffected.

In several studies, a high ratio of P:Ca (approximately 3–4:1) in conjunction with low Ca intake led to adverse effects on bone health. Many of these reports had small numbers of subjects or were of short duration. For example, a cross-sectional study of 38 young females found a negative association between P intake and radial bone measurements [9]. Meaningful data interpretation was unlikely with such a small sample. A cross-sectional observational study of 147 healthy females aged 31–43 years by Kemi et al. [6,7] reported that with adequate Ca intake, the lowest quartile (Ca:P ratio of <0.51) had higher mean serum parathyroid hormone (S-PTH) concentration (P = 0.021) and mean urinary Ca (U-Ca) excretion (P = 0.051) than all other quartiles. It should be noted that the number of subjects in the study was too small for an observational study to make a reliable conclusion. Randomized clinical trials have shown that the combination of high P (1,500-1,700 mg) coupled with low Ca intake (375–400 mg) increased serum PTH concentration in healthy young females [4,5,8]. Portale et al. [10] found that a high intake of P decreased the serum calcitriol concentration in healthy males. It also acutely inhibited bone formation [4].

Kemi et al. [18] reported that when P intake was above current recommendations, increased Ca intake was beneficial for bone, as indicated by decreased S-PTH concentration and bone resorption; not even high Ca intake had effect on bone formation when P intake was excessive. In this study, 12 healthy female subjects aged 21–40 years attended three 24-h study sessions. They were randomized according to a Ca dose of 0 (control day), 600, or 1,200 mg, and each subject served as her own control. The meals on each study day provided 1,850 mg P and 480 mg Ca. Rising Ca doses were accompanied by decreasing concentrations of serum PTH (P < 0.001) and increasing serum ionized Ca concentrations (P < 0.001), with no significant differences in the bone formation marker and serum bone-specific alkaline phosphatase. It is worth noting that the study duration of this study was too short to see a meaningful impact on bone health parameters.

In the U.S., mineral intake is generally adequate. The IOM [3] stated that in balance studies, Ca:P molar ratios of 0.08-2.4:1 (a 30 fold range) had no effect on either Ca balance or absorption. The IOM also found that the intake ratio alone failed to take into account physiological adaptive responses, such as bioavailability and renal output. For example, in term-born infants during the first year of life, a higher Ca content in soy-based formulas reduced P absorption, but retention was similar due to offsetting changes in renal P output. Thus, the IOM concluded that there is little evidence for relating the two nutrients.

Adequate dietary P is essential for building bone since bone mineral is predominantly calcium phosphate [3]. Milk is the leading source of dietary P in the American diet, but a synergistic effect of other nutrients in milk (e.g., calcium, protein, potassium, magnesium) may improve absorption of dairy Ca [14]. Although Ca supplements reduce its absorption compared to dairy milk [14], those that contain P (e.g., tricalcium phosphate, dicalcium phosphate) maintain strong bones and reduce the risk of osteoporosis [15]. Our data show that high P intake is associated with high Ca intake as well as improved bone health in some age/gender groups.

Other findings demonstrate beneficial effects of Ca phosphate supplementation. Anabolic agents for treating osteoporosis require positive P balances of up to 90 mg/day [19]. A Ca phosphate supplement may be preferable to carbonate or citrate salts because it preserves food P [15]. Ditscheid et al. [20] reported that pentacalcium hydroxy-triphosphate (Ca_5_(PO_4_)_3_OH) supplementation provided additional daily intake of 1060 mg Ca and 490 mg P. These amounts significantly lowered serum total and LDL cholesterol concentrations by 6.5% and 3.6%, respectively, after 4 weeks of supplementation.

This study has several shortcomings. First, its cross-sectional design precludes the assignment of cause and effect. A prospective, longitudinal study may be more appropriate for a population study. Second, lack of PTH data in the NHANES dataset did not allow analysis of the impact of high P intake on blood PTH concentrations. Additional studies with parameters beyond bone health should be considered before increasing P consumption.

## Conclusion

Our cross-sectional analyses of NHANES data show that high P intake is associated with a 4.2% improvement in BMC and a 2.1% improvement in BMD. It also reduced risk of osteoporosis by 45% in adults whose Ca and P intakes were within normal ranges.
